# A fresh perspective on infrared spectroscopy as a prescreening method for molecular and stable isotopes analyses on ancient human bones

**DOI:** 10.1038/s41598-024-51518-5

**Published:** 2024-01-10

**Authors:** Cinzia Scaggion, Maurizio Marinato, Gregorio Dal Sasso, Luca Nodari, Tina Saupe, Serena Aneli, Luca Pagani, Christiana L. Scheib, Manuel Rigo, Gilberto Artioli

**Affiliations:** 1 Department of Geosciences, University of Padova, 35131 Padova, Italy; 2 INSTM, National Interuniversity Consortium of Materials Science and Technology, 50121 Firenze, Italy; 3 Department of Cultural Heritage: Archaeology and History of Art, Cinema and Music, University of Padova, 35139 Padova, Italy; 4 Institute of Geosciences and Earth Resources, Italian National Research Council-CNR, 35131 Padova, Italy; 5 Institute of Condensed Matter Chemistry and Technologies for Energy, Italian National Research Council-CNR, 35127 Padova, Italy; 6 Estonian Biocentre, Institute of Genomics, University of Tartu, 51010 Tartu, Estonia; 7 Department of Biology, University of Padova, 35122 Padova, Italy; 8 Department of Public Health Sciences and Pediatrics, University of Torino, 10126 Torino, Italy

**Keywords:** Environmental sciences, Environmental social sciences

## Abstract

Following the development of modern genome sequencing technologies, the investigation of museum osteological finds is increasingly informative and popular. Viable protocols to help preserve these collections from exceedingly invasive analyses, would allow greater access to the specimens for scientific research. The main aim of this work is to survey skeletal tissues, specifically petrous bones and roots of teeth, using infrared spectroscopy as a prescreening method to assess the bone quality for molecular analyses. This approach could overcome the major problem of identifying useful genetic material in archaeological bone collections without resorting to demanding, time consuming and expensive laboratory studies. A minimally invasive sampling of archaeological bones was developed and bone structural and compositional changes were examined, linking isotopic and genetic data to infrared spectra. The predictive model based on Infrared parameters is effective in determining the occurrence of ancient DNA (aDNA); however, the quality/quantity of aDNA cannot be determined because of the influence of environmental and local factors experienced by the examined bones during the burial period.

## Introduction

Osteoarchaeological remains are often the only remaining physical evidence of the presence of humans and animals, containing valuable biological information about the past. In particular, the dense bone of the *pars petrosa* and of the tooth roots can provide a high concentration of endogenous ancient DNA (aDNA)^1–3^. Due to their unique informational value, for forensic/medical sciences, exhibition purposes, isotopic and evolutionary studies, such tissues are the most used sample source. Bones and teeth are calcified tissues characterised by a complex hierarchical structure from the macro- to the nano-scale and mainly consist of mineral and organic phases^4,5^. The inorganic component, bioapatite, is a defective nanocrystalline carbonated-hydroxylapatite phase with a chemical composition significantly departing from the stoichiometric hydroxylapatite Ca_5_(PO_4_)_3_(OH) and constitutes about 60% of bone. 30% of bone material is constituted by organic components such as collagen, protein-mucopolysaccharide complexes and glycoproteins, while the remaining 10% is structural water^6^. Collagen fibrils (the majority is Type I collagen that accounts ca. 90% of the organic fraction of bone) is the major fibrous protein which gives elasticity and tensile strength to the bone system^7^ and is the one of the primary components for biomolecular investigations such as radiocarbon dating, stable isotopes and genetic analyses for diet reconstruction, mobility and species identification. Deoxyribonucleic acid (DNA) in living bone is located within several cell types, such as preosteoblast, osteoblast, osteoclast and osteocyte^8^. Osteocytes, typical of mature bone, represent 90% of all bone cells and are enclosed in a niche carved into the intracellular bone substance, called *lacunae*^8^*.* Tooth cementum is rich in cementocytes, which are DNA-containing cells that remain encased in the mineral structure of the tooth^9,10^.

*Post-mortem* alterations result from the combination of different processes that affect the organic and inorganic components of bone, degrading them^11^. Humidity, temperature, microbial activity, soil composition, pH, and circulating water affect bone preservation. In particular, collagen in the presence of alkaline pH undergoes hydrolysis^12–14^, while acidic pH promotes the dissolution and/or recrystallization of bioapatite^15^ with possible incorporation of exogenous ions into the crystalline structure. Subsequently to bioapatite dissolution, the organic component is exposed to microbial attack, fungi and bacteria that cause hydrolysis and leaching^12,16–20^*.* Variations in the climatic and environmental conditions of the burial affect the stability of the bone constituents, causing gelatinization of collagen, intensification of microbial and enzymatic activity with consequent dissolution/reprecipitation of the mineral phase and demineralization of the bone^12,16, 21^. Diagenetic changes alter the microstructure of bone causing chemical alterations, unfavorable for DNA survival^22,23^ while progressively reducing the content of organic components in ancient samples (> 1% of endogenous DNA content), making analysis on a genomic scale impossible and/or very expensive^24–26^. At the ultrastructural level, DNA can establish strong bonds with both the organic and the inorganic components, so that the adsorption of DNA to the surface of bioapatite nanocrystals as well as the linking with Type I collagen may stabilise it and may determine its outliving over time^13,27, 28^. Genetic molecules can bind strongly to both organic and inorganic bone phases; therefore, an extraction method to recover double helix molecules is applied to both fractions, with a higher chance of success, employing EDTA (to demineralise the bioapatite) and Proteinase K (to digest osseous proteins)^29^. Furthermore, in archaeological contexts, there is a very high site-specific variability in the quality/quantity of DNA recovered from bones, even when the samples originate from similar burial environments and show very similar preservation states, as highlighted in genetic research^30,31^.

Sampling methods for ancient DNA extraction are predominantly destructive, causing the loss of the entire root, the drilling and cutting of the *cochlea* (located in the petrous portion of the temporal bone), to powdering or coring the bone findings^32^. Some bones survive well, while others degrade rapidly; and since it is difficult to evaluate a *priori* the preservation of organic biomolecules within a bone, expensive and often destructive analyses have been the norm to assess the suitability of a given sample to be included in molecular studies, so far^33^.

The application of prescreening methods can overcome the major problem of identifying suitable materials in archaeological bone collections. Tooth and petrous bone sampling can be destructive, particularly when only one or a few of them are preserved (e.g. in museum collections or ossuaries). Moreover, invasive sampling may cause the decrease of the expositional value and may hamper morphological studies that provide important information about the age estimate of an individual for forensic and anthropological cases, but also the diet, cultural habits, and evolution^34–39^. Therefore, before irreversibly damaging precious materials, the sample choice has to be based on solid evidence on the positive outcome of a planned analysis, so that pros and cons can be evaluated by interacting with museum curators, conservators and archaeologists^40^. Recent methodological advances in several research fields have increased the request for sampling from museum collections and raised ethical concerns over the destruction of human remains^32,41^. Currently, several strategies have been adopted to protect the long-term integrity of collections by devising minimally invasive sampling protocols^42–44^.

Different methods and tools were used to reduce the destructive impact of sampling for DNA extraction and, in this perspective, numerous studies have been carried out to design a minimally invasive protocol to safeguard osteological findings until prescreening of bone preservation^28,45−62^. In previous studies, the evaluation of new parameters capable of identifying samples that may contain original sequences led to a focus on proteins; the degree of racemization of amino acids was then examined as a proxy^46,54,55,63–66^, being a minimally invasive technique (less than 10 mg of bone are required). Diagenetic changes and the amount of preserved collagen have been examined in relation to the success or failure of nuclear DNA amplification through several analytical and spectroscopic methods^28,47,49,50,52,53,67^, determining that hydroxyapatite plays a crucial role in DNA preservation and that the organic component may indicate its presence.

Within this context, this work aims at developing a molecular prescreening method through the direct evaluation of the degree of *postmortem* recrystallization of the bioapatite and correlating it with the quality/quantity of collagen, that could allow a more accurate selection of well-preserved bone samples, reducing the costs of genetic analysis. We investigate the samples variability in terms of preservation state through the micro-sampling of osteological findings, characterised by different chronology and origin. Comparing bone integrity with the data obtained from the genetic analysis is an essential step for developing a prescreening method, applicable to all osteoarchaeological findings. In this work, the technique of choice is Fourier-transform infrared spectroscopy (FTIR spectroscopy), which is an advantageous technique, providing a fast, convenient, minimally invasive and accurate semi-quantitative method to investigate both the organic and inorganic components of mineralised tissues. Moreover, FTIR has been successfully applied to in-depth studies on the alteration of teeth and bones caused by diagenetic processes^16,68–71^. Infrared absorption bands are characteristic of specific types of chemical bonds, enabling the description of variations on the structural properties of organic and inorganic components^72^ to assess bone quality and exploring sample variability.

Here, we intend to propose a prescreening method to investigate the relationship between the characteristic features of FTIR spectra with the amount of DNA contained in the bone. Since the survival of DNA is bounded by multiple inter correlations, the study is integrated with the examination of diagenesis-induced microstructural modifications in the secondary structure of the preserved collagen^49,53,73^. Organic preservation was investigated by analysing the infrared spectra of lyophilized collagen to determine its integrity, examining in detail its characteristic FTIR peaks^74–76^. The results of FTIR analysis were compared with aDNA yields and C:N atomic ratio to assess whether the collagen quantity and, in particular, quality can represent a diagnostic proxy for genetic extraction. Here, we aim at improving the selection process of bone samples for different fields of interest, such as stable isotopes and radiocarbon analyses by using predictive parameters based on micro-invasive FTIR spectroscopy.

## Results

### Measurements of the infrared spectra of petrous bones and tooth roots

**Figure 1:**
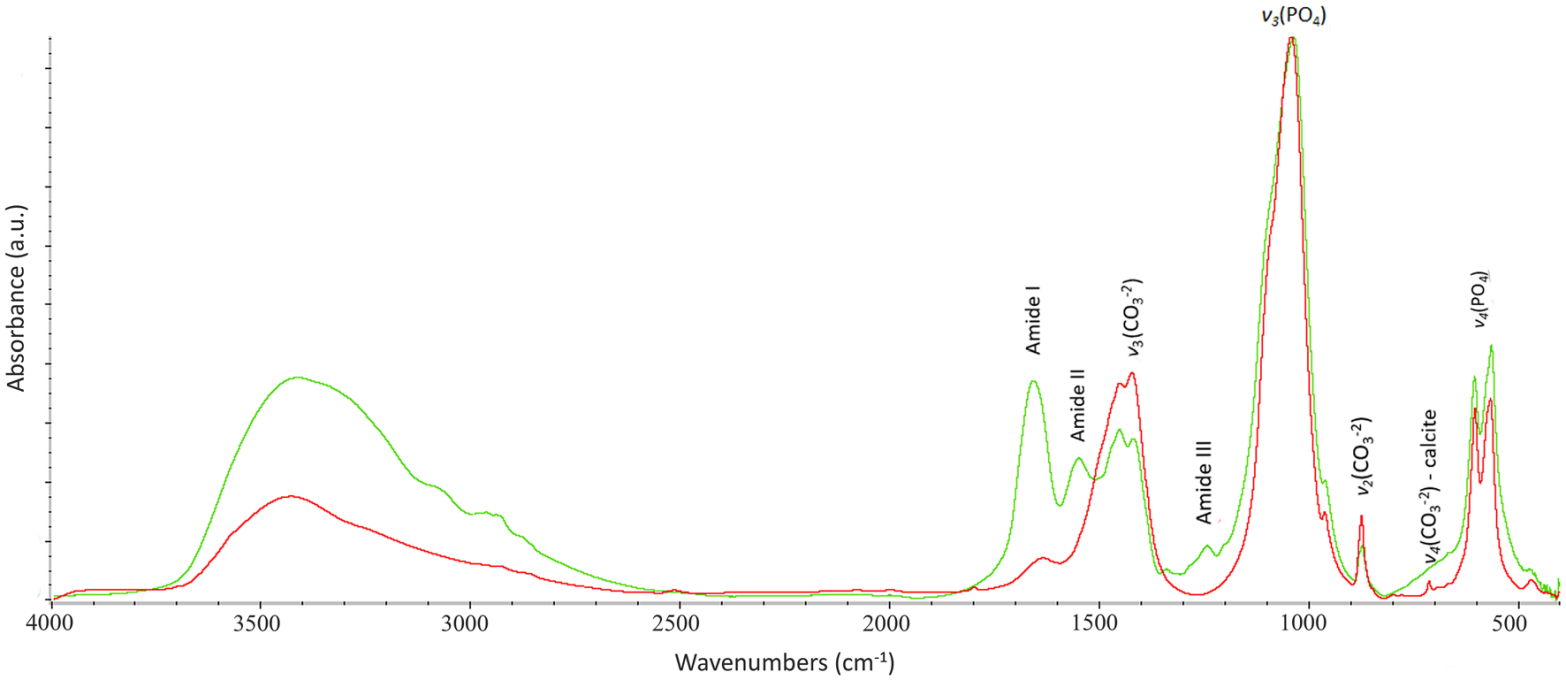
FTIR spectra of two bone samples showing the characteristic vibrational bands attributable to the amide, phosphate and carbonate groups. The two spectra reflect the different states of preservation of bone samples: a well-preserved bone from a stable climate (cave) environment (green line) and a poorly preserved bone from temperate environment (red line).

IR absorption bands are characteristic of specific chemical bonds and provide insights on the nature and on the structural properties of the organic and inorganic components that make up the bone at molecular scale^72,77^. The spectrum of bone shows the characteristic band distribution attributable to the amides, phosphates, and carbonate groups. Figure 1 shows the different FTIR spectral features occurring in well-preserved (green line) and poorly preserved (red line) bones. Qualitative and semi-quantitative analyses can be performed directly on FTIR spectra by calculating suitable parameters based on the the intensity ratio and width of FTIR peaks.

All acquired bone FTIR spectra display a broad band in the range 3500–2500 cm^−1^ generated by ν_1_ and ν_3_ stretching modes of hydrogen-bonded (H–O–H bonds) water molecules and broad resonance doublet of the hydrogen bond at ~ 3310–3270 cm^−1^ and at ~ 3100–3030 cm^−1^ related to Amide A and Amide B (N–H stretching), proteins insensitive to the conformation of the polypeptide backbone^78^.

Vibrational modes are evident in the region between 2961 and 2925 cm^−1^ and between 2875 and 2850 cm^−1^, corresponding to the organic compounds of the bone system, attributed to asymmetric stretching C–H_3_ and C–H_2_ bonds in the aliphatic chains of collagen, respectively^78^.

Moving the focus to the fingerprint region (1800–400 cm^−1^), specific collagen absorption bands were identified and related to C=O stretching vibrations, N–H bonding and the coupling of the C–N stretch to the N–H bend, corresponding to Amide I (1660 cm^−1^), Amide II (1550 cm^−1^) and Amide III (at 1340–1240 cm^−1^), respectively. Notably, in the Amide I region the superimposed contribution of ν_2_ (O–H) at 1645 cm^−1^ bending mode of the structural water occurs^79^.

The bone powders show the ν_3_(CO_3_^2−^) asymmetric stretching peak at 1452–1425 cm^−1^ and the ν_2_(CO_3_^2−^) out of plane bend, at 875 cm^−1^ of structural carbonate^80^. For all spectra, the contribution of the ν_3_(PO_4_^3−^) band at 1090 cm^−1^, due to the asymmetric vibration of the phosphate ions, appears as a subtle shoulder, indicating a more ordered bioapatite when it is more evident^81,82^. The absorption bands at 1035, 604 and 565 cm^−1^ correspond to the asymmetric stretching and bending (ν_3_, ν_4_) of phosphates, while a weak band at 960 cm^−1^ is attributed to the ν_1_(PO_4_^3−^) symmetric stretching mode. About half of the samples analysed show the ν_4_(CO_3_^2−^) peak of the in-plane bending vibrational mode at 712 cm^−1^ which is characteristic of calcite, occurring as a secondary mineral phases in archaeological bones.

### FTIR measurement of lyophilized bone collagen and quality control (QC).

**Figure 2:**
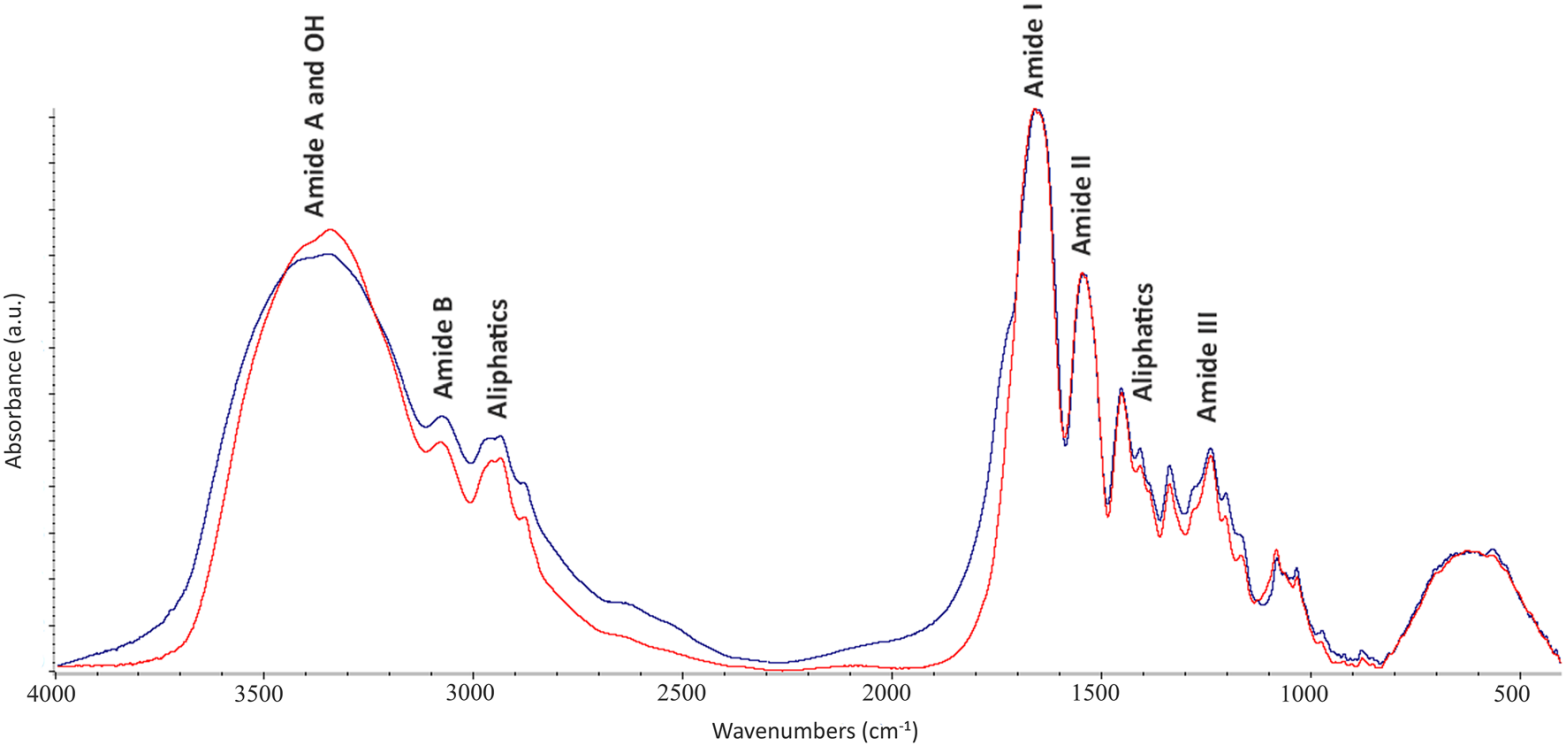
FTIR spectra of collagen extracted from an archaeological tooth root (blue line) and a modern bone sample (red line), showing the characteristic vibrational bands of collagen. Different FTIR features can be observed between the archaeological (blue) and modern (red) samples.

Spectra shows the characteristic band distribution of collagen in the regions 3500–2800 cm^−1^ and 1800–800 cm^−1^ (Fig. 2).

All spectra present absorptions at 3500–3300 cm^−1^ related to Amide A (N–H str.) and O–H vibrations, at 3079 cm^−1^ assigned to Amide B (N–H str.) while those at ~ 2970 and 2935 cm^−1^ are assigned to long-chain linear aliphatic compounds (CH str. and CH_3_ str.)^83,84^. Characteristic absorption bands of Amide I (1660 cm^−1^), Amide II (1550 cm^−1^), aliphatic compounds (1450–1407–1385–1338 cm^−1^) and aromatic (1280 cm^−1^), Amide III (1238 cm^−1^) and carbohydrates (from 1200 cm^−1^ to 874 cm^−1^) were identified^78,83,85,86^. The sample DSL17-D_coll (collagen from petrous bone sample) shows a strong vibrational mode at 3464 cm^−1^ (–OH groups str.) and a shift in the Amide I region from 1660 to 1645 cm^−1^. All collagen spectra of archaeological bones show a weakly defined shoulder at 1720 cm^−1^, whereas this is not present in the collagen extracted from animal fresh bone.

The area ratio of the infrared underlying bands of the Amide I band, 1660:1690 cm^−1^, which provides information on the maturity of collagen linked to enzymatic cross-linking, ranges from 1.57 to 1.51 for Grottina Covolini del Broion, from 1.58 to 1.36 for Ordona, from 1.65 to 1.49, for Salapia, from 1.20 to 1.16 for Desenzano del Garda, and from 1.99 to 1.61 for animal fresh bone and San Giovanni Rotondo, respectively.

All lyophilized collagen samples previously analysed by FTIR were subjected to the quality control criterion (QC) for stable isotopes analysis by measuring the carbon-to-nitrogen (C:N) atomic ratio in order to verify their state of preservation (all information are reported in Material and Methods). Covoloni del Broion, Ordona, Salapia, and San Giovanni Rotondo samples provided values falling within the range of well-preserved collagen (ranging between 2.9 and 3.6^87^), while samples from Desenzano del Garda (signed DSL) show an off-scale C:N ratio, resulting in a poor preservation which invalidated the stable isotopes analyses.

All data are reported in Supplementary Table S1.

### FTIR data analysis compared with endogenous aDNA yields

The mineral-to-matrix indicator (AmI/PO_4_^3−^), proportional to the amount of preserved collagen^88^, has been measured obtaining high variations between samples with values ranging from 0.11 to 0.40 for Grottina Covoloni del Broion samples and from 0.05 to 0.19, from 0.07 to 0.40 and from 0.10 to 0.27 for Ordona, Salapia and San Giovanni Rotondo, respectively. AmI/PO_4_^3−^ values range from 0.08 to 0.33 for Desenzano del Garda samples while a value of 0.39 was measured for the ox bone.

AmII/PO_4_^3−^ parameter shows values ranging from 0.10 to 0.25, from 0.06 to 0.17, from 0.04 to 0.14 and from 0.07 to 0.16 for Covoloni del Broion, Ordona, Salapia and San Giovanni Rotondo, respectively, while values from 0.07 to 0.20 were obtained for Desenzano del Garda and a value of 0.24 for the ox bone (Table S1).

Table S1 shows the FW85% values for all samples, ranging from 8.67 to 12.51 cm^−1^ and the corresponding IRSF values, ranging from 5.13 to 3.01. Dividing the samples by origin, large variations are evident in Desenzano del Garda samples with values from 9.40 to 11.21 of FW85% with IRSF from 4.25 to 3.14 and for San Giovanni Rotondo with FW85% from 9.28 to 11.44 and IRSF of 3.81 to 3.27. The set of cave samples is divided into two sub-sets with variations in phosphate peak width ranging from 9.40 to 10.46 and IRSF between 3.76 and 3.41 while the other subset comprises values ranging from 11.20 to 12.25 and 3.53 and 2.99 for FW85% and IRSF, respectively. A high variability in the yield of ancient DNA (%) is shown for the sequenced samples. For Broion samples, endogenous DNA yields range from 0.07% to 17.32%, while larger amounts were found in the Oderzo samples between 0.69% and 43.75%. High yields of sequenceable endogenous DNA were not obtained for Salapia and San Giovanni Rotondo, providing aDNA content ranging from 0.15 to 4.71% and 3.72 and 6.39%, respectively. Of these samples, only SGR002 (San Giovanni Rotondo) and SAL003 (Salapia) contain high yields of aDNA with 17.79% and 28.16%, respectively. All the data obtained from the genetic analysis are shown in Table S1.

### Statistical analysis

In Table 1 the values of the intercept and the slope (coefficients) for endogenous aDNA and *p*-values for the coefficient are reported.

**Table 1:** Simple regression model in R software statistics using FW85% as independent variable.

Linear Regression Equation: aDNA% = − 98.0698 + 11.2778 FW85%									
Term	Coef	SE Coef	T-Value	*P*-value	S	R-sq	R-sq(adj)	F	df
Intercept	− 98.0	10.1	− 9.6	1.01e-15					
FW85%	11.2	0.9	11.3	< 2e-16	20.69	0.5754	0.571	128.8	1,95

The linear model using the FW85% parameter provided the best fit for aDNA yield, with a *p*-value < 2*10^−16^ and R^2^ = 0.58. Among the considered variables, FW85% provided the most significant model, with a p-value of 5.16*10^−5^ (Table 1).

## Discussion

The preservation state of osteoarchaeological human samples here analysed was investigated by considering a large set of samples previously characterised (see Scaggion et al.,^89^) taking into account a large and diverse chronology and origin with high variability in terms of preservation state due to different diagenetic processes occurring within the burial environments.

The samples, subjected to different environments, were selected to evaluate whether the prescreening method here proposed could be considered of general applicability. The same bone elements, such as tooth roots and petrous bones, were examined in each selected necropolis, enabling a prompt a comparison of results. The width at 85% of the height of the ν_4_(PO_4_^3−^) vibrational mode at 604 cm^−1^ (FW85%) was chosen as it produces a well-defined signal that is less influenced by other overlapping vibrational modes, describing the variation of bioapatite structural properties through FTIR spectroscopy^81^. This parameter is useful for tracking subtle variations in terms of physico-chemical properties in well-preserved samples as well as in highly diagenetically altered samples.

**Figure 3:**
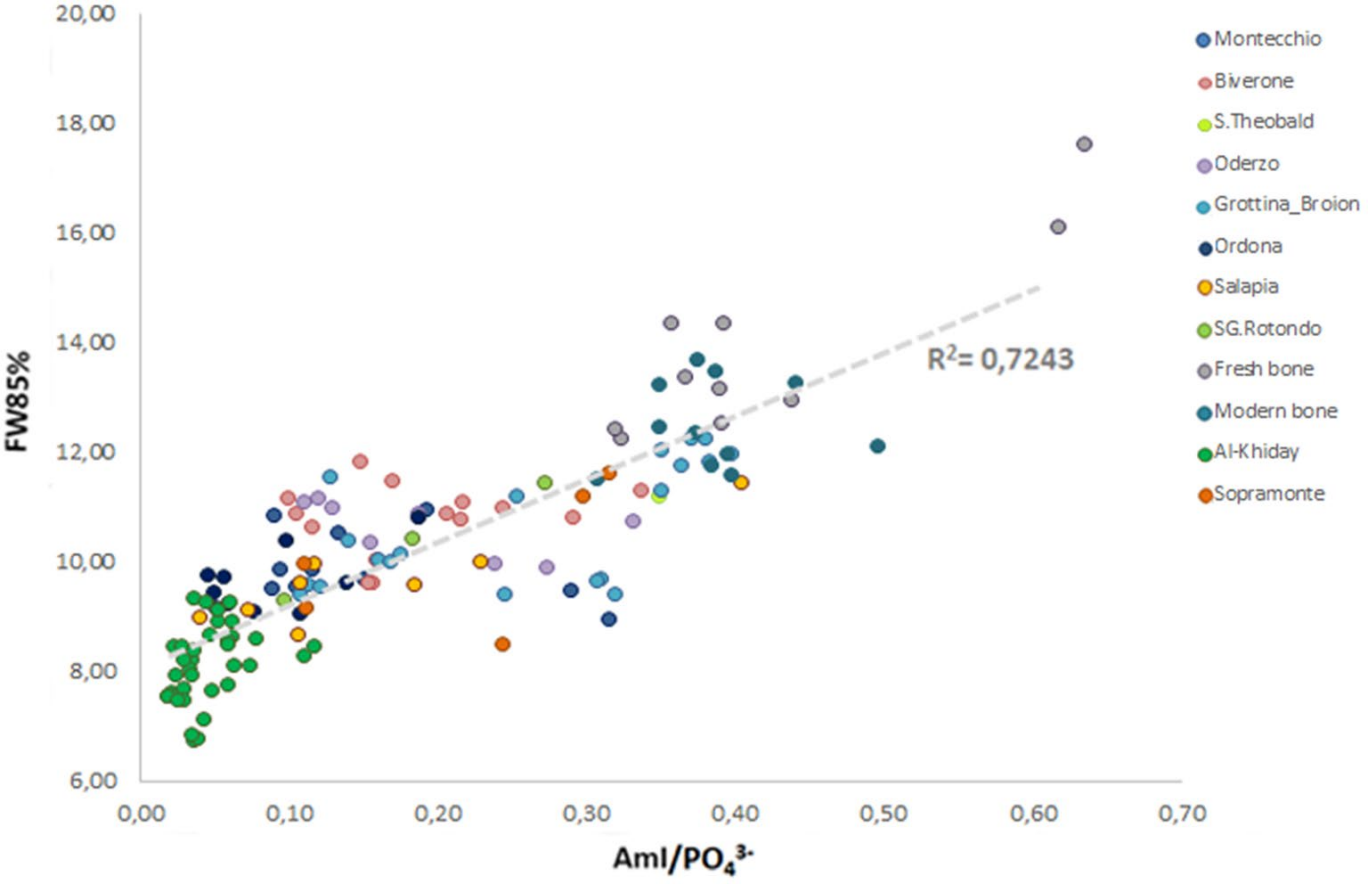
FTIR data, reported in Scaggion et al.^89^, show the correlation between AmI/PO_4_^3−^ and FW85%.

AmI/PO_4_^3−^ is widely used to quantify the presence of collagen preserved in the bone system, nevertheless the water signal overlaps to the amide band. The width of the phosphate peak indirectly depends on the collagen content, as also confirmed by the AmI/PO_4_^3−^ - FW85% correlation, since the presence of collagenous material in the hierarchical structure of the bone plays an important role in controlling the crystallite size of bioapatite after the death of the individual. The data provided by these two parameters enable one to effectively determine the degree of alteration of the examined samples (Fig. 3).

Samples exhibiting high values of FW85% (higher than 8.96) and collagen content (higher than 0.04) were considered promising for DNA extraction. All selected samples produced sequenceable aDNA but with high variability in terms of endogenous DNA yields. Besides FTIR spectroscopy, collagen quality is commonly assessed by the measurement of the C:N atomic ratio. Despite the same environmental conditions, genetic analysis, carried out on the selected samples, revealed that samples with similar preservation states, as determined by FTIR parameters, and well preserved in terms of C:N ratio, produced different yields of aDNA. To ascertain whether the quality of collagen was indicative of the amount of extractable aDNA, the FTIR spectral analysis was performed on the collagen extracted from the samples that provided a good C:N ratio and a relevant amount of aDNA. Three medieval samples coming from the Desenzano del Garda site, characterized by C:N ratios not meeting the quality criterion, were selected for comparison to determine the effectiveness of 1660:1690 cm^−1^ infrared parameter in defining the quality of collagen in terms of secondary structure. The collagen secondary structure was investigated by analysing the Amide I band (sensitive to the backbone of secondary structure^90^) through spectral deconvolution, measuring the two sub-bands ratio 1660:1690 cm^−1^. The Type I collagen consists of a triple helix made of the repetitive amino acid sequence glycine-X–Y, where X and Y are frequently proline or hydroxyproline and constitute the primary structure, called α-chains^91,92^. Polypeptide chains are linked to other chains through hydrogen bonds, which lead to the formation of the secondary structure, which can be of two types: α-helix and β-sheets^90^. FTIR spectroscopy provides information on the secondary structure content of proteins.

**Figure 4:**
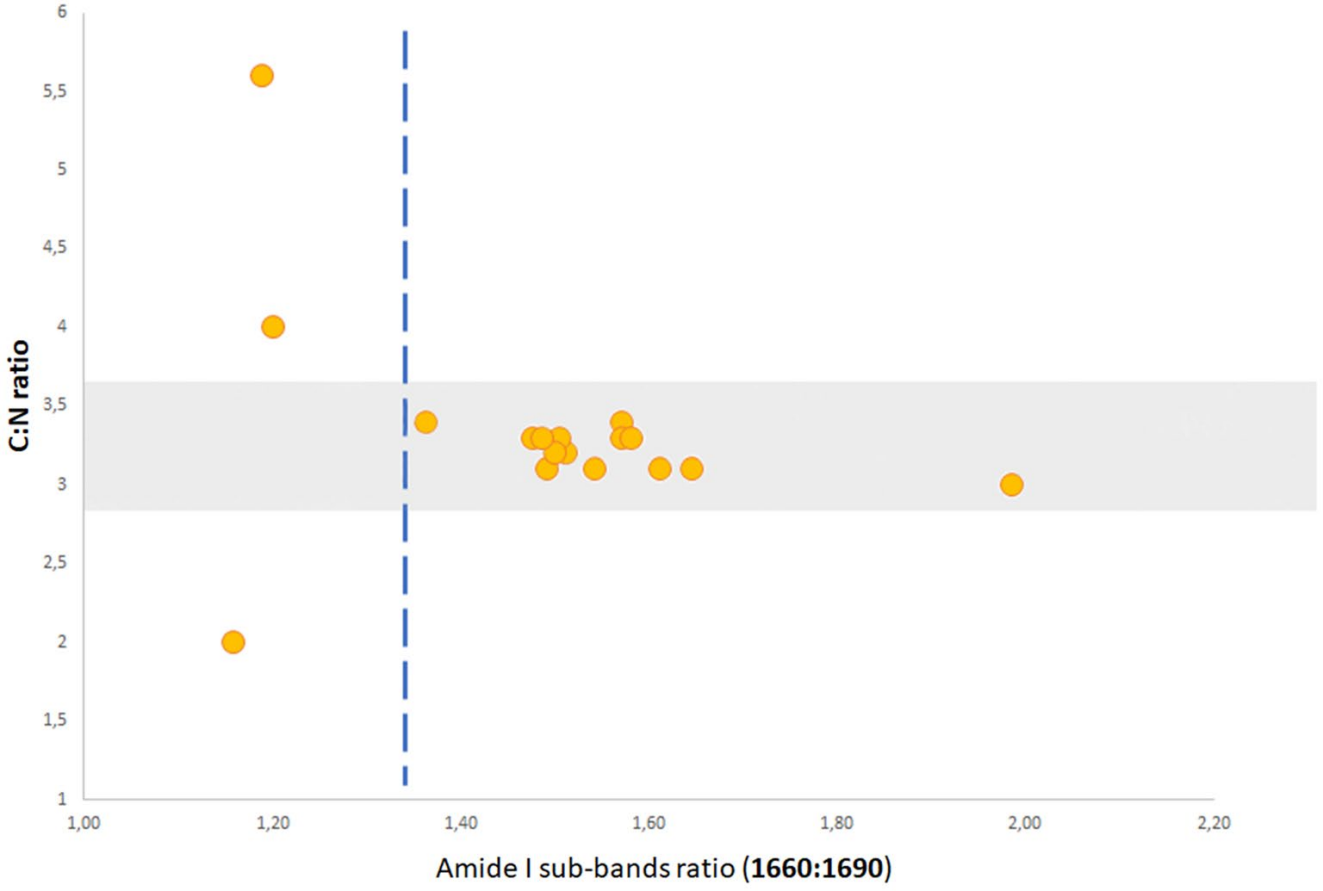
Comparison between the C:N ratio and 1660:1690 cm^−1^. Below the threshold value, indicated by the dashed blue line, the quality of collagen determined by the C:N criterion reveals the relationship with the alteration of the secondary structure of collagen by the ratio of the main two Amide I sub-bands. The figure highlights that above the value of 1.30, obtained from the ratio between 1660:1690 cm^−1^, the collagen samples show C:N atomic ratios within the 2.9–3.6 range, meaning a good preservation state.

The characteristic bands found in the infrared spectra of proteins and polypeptide chains are Amide I and Amide II. These arise from the amide bonds that connect amino acids. The absorption of the Amide I band is associated with the stretching vibrations of the C=O bond of the amide; the absorption of the Amide II band is mainly associated with the bending vibrations of the N–H bond. Since both C=O and N–H bonds are involved in the hydrogen bonding that occurs between the different elements of the secondary structure, the positions of both the Amide I and Amide II bands are sensitive to the content of the secondary structure of a protein^93^. Studies with proteins of known structure were used to systematically correlate the shape of the Amide I band with the secondary structure of proteins^94,95^. Molecular spectroscopy is useful to infer the quality of collagen^52,73,75,96–99^ and to that end, the Amide I (1660 cm^−1^) vibrational band is uniquely informative. The changes in the Amide I profile with denaturation may reflect alterations in the collagen secondary structure, specifically a transition from the ordered to the less-ordered structure. The main degradation processes of collagen are oxidation, hydrolysis, and denaturation^12^. All FTIR spectra of lyophilized collagen from archaeological bones show a weak shoulder at ~ 1720 cm^−1^, associated with acidic carboxyl groups, probably linked to a process of hydrolysis by soil humic acids^100,101^. Increased protein carboxylation is associated with collagen protein fragmentation and aggregation. In this perspective, the FTIR band ratio 1660:1690 cm^−1^, related to the maturity of collagen cross-links^102^, is negatively correlated with collagen quality: the lower the ratio between these band areas, the higher the degenerative state of collagen^84^. This negative correlation, associated with the presence of the vibrational band of the carbonyl compounds, was confirmed by comparison between the 1660:1690 cm^−1^ ratio and the C:N data (Fig. 4).

Based on the results obtained from this set of samples, it is possible to estimate that below a certain threshold of approximately 1.30 (1660:1690 area ratio) (Fig. 4) the value of the 1660:1690 cm^−1^ ratio indicates instability in the collagen structure and a consequently poor preservation.

As the deterioration proceeds, covalent bonds are cleaved and collagen molecules become unstable, leading to gelatinisation and the irreversible denaturation of polypeptide chains^103^. Secondary collagen structure could be used as a prescreening tool for paleo-diet studies or radiocarbon dating, while no correlation was found with aDNA yields.

By evaluating the spectra of archaeological bones, the shoulder linked to the denaturation of collagen (~ 1720 cm^−1^) is not visible, probably due to the overlap with other vibrational bands of organic substances from the archaeological context (humic acids, fulvic acids)^104^. Furthermore, the application of the ratio between the two amide sub-bands at 1660 cm^−1^ and 1690 cm^−1^ as a prescreening method directly on bone spectra is not straightforward due to the band overlap.

On the other hand, the statistical analysis on the FW85% parameter in correlation with the occurrence of aDNA provided more promising results. The overall quality of the linear regression fit can be assessed using the Residual Standard Error (RSE = 20.69), displayed in the model summary (Table 1), indicating that the model fits well the data.

This model suggests that the parameter related to the inorganic component (FW85%) has predictive potential for the presence/absence of aDNA. These results are in agreement with recently published research, confirming a more intimate association between DNA and inorganic components, with an interplay between DNA and the bioapatite crystal growth and morphology^105,106^. Indeed, it has been highlighted that DNA molecules have high affinity for binding to bioapatite crystals, so that its survival in calcified bone may be determined by phosphate-promoted molecule desorption, suggesting an ion exchange process between phosphate anionic groups of the DNA backbone and non-apatitic hydrogen-phosphate ions released from the hydrated layer of synthetic biomimetic apatite.

In conclusion, FW85% infrared parameter could be a promising tool to evaluate the extraction potential of genetic molecules from archaeological bone samples.

## Conclusion

The results discussed here show that the predictive model based on the inorganic fraction of bones and teeth is effective for aDNA occurrence; however, the quality/quantity of aDNA cannot be determined by the analysis of secondary structures of the organic component. The quantity/quality of endogenous DNA may not be predictable because of the influence of environmental local factors to which bones were subjected during burial. Measuring the FTIR signals of the phosphate peak width may predict the occurrence of aDNA, but it is still difficult to fully describe a multifactorial process.

Indeed, the relationship between DNA and bioapatite and how their chemical interaction is functional to the molecular preservation during diagenetic processes need further investigation, for the benefit of molecular-anthropological and forensic studies.

Besides the aDNA analysis, FTIR can be profitably used as a prescreening method for C and N stable isotopes analyses. At first, FTIR can be directly applied to bone specimens to determine the preservation state of both the organic and inorganic components. Subsequently, if the quantity of collagen is considered sufficient for extraction, the secondary polypeptide structure can be analysed in-depth before proceeding with further laboratory analyses.

## Materials and methods

### Bone samples

In this study, samples were selected from human skeletal remains (more precisely, petrous bones (n = 20) and tooth roots (n = 24)) covering different chronologies and sites, presenting different preservation states curated at the Museum of Nature and Humankind-Anthropology section of the University of Padua, Italy. The analysed bones came from graves in Northern and Southern Italy (Covoloni del Broion, Salapia, Ordona, San Giovanni Rotondo, and Desenzano del Garda). The stratigraphy of the Covoloni del Broion cave, highlighted by systematic excavations, consisted of thin silty layers of terrain interspersed in stalagmite concretions, incorporating human skeletal remains of the Bronze Age^31^, followed by a series of silty layers with abundant crushed stone, partially concreted^55^. The Daunian necropolises of Salapia^107^ and Ordona are located at 10 and 20 km from contemporary Cerignola (Foggia, Italy), respectively, while there are no archaeological records on the nearby San Giovanni Rotondo. The necropolis of Ordona was the subject of two archaeological campaigns in 1978 and 1981^107^ and is characterised by a chronological continuity in the burials that includes the Iron Age up to the Roman Empire. After a period of neglect, the use of the necropolis was resumed in the Middle Ages. Despite the lack of archaeological information, recent studies have confirmed the attribution to the Iron Age of most of the graves^30^. Three bone elements of early medieval origin come from the site of the Church of San Lorenzo in Desenzano del Garda (Brescia, Italy) in agricultural territory (more information can be found in Canci et al.,^108^). Data of ancient samples were compared with those obtained from fresh animal bones. The list of bone samples sequenced and analysed by FTIR spectroscopy and mass spectrometry is reported in Table S1.

### Micro-sampling for Infrared analysis

The external surface of all bones and roots surface was mechanically removed by means of a low-speed micro-drill, equipped with an abrasive round point of 2.4 mm using a Dremel micro-tool. The procedure was carried out to avoid contamination of exogenous material, that could invalidate the spectral analyses. The powdered material from the tooth roots and petrous bones was extracted with a round engraver bit (2.4 mm diameter) after removal of the external deposits, while on other occasions for genetic analysis a complete root was chosen and cut with a cutting disc wheel (diameter 20 mm). The drill bits used for sampling operations were sterilised to avoid external contamination, using the procedure for standard sampling of bone of the ICMP (International Commission on Missing Persons—ICMP 2015) was followed.

### Sample preparation and FTIR spectra collection

Enough bone material was extracted from the petrous bones and tooth roots (20–30 mg) to perform the FTIR analysis. Pellets of bone powder and lyophilized collagen were prepared, maintaining a ratio 1:100 mg of sample/KBr with constant grinding for 2 min. The powder obtained was then pressed, using a hydraulic press, under 11 tons/cm^2^ pressure, and a transparent pellet of 12 mm in diameter and 1.5 mm in thickness was obtained. Spectrum was collected with a Nicolet iS 10 FTIR spectrometer equipped with a DTGS detector; 128 scans were acquired, in the range from 4000 to 400 cm^−1^, with a spectral resolution of 4 cm^−1^. Spectral analysis was performed using Omnic 9 software (Thermo Scientific).

### Bone spectra analysis

Spectral analysis was performed for all spectra; the peak heights of Amide I and of the ν_3,_ ν_4_ vibrational modes of phosphate were selected and calculated from the baseline defined by two points calculated as the local minimum ranging in selected regions of the spectrum: 2000–1800/1400–1200 cm^−1^, 1400–1200/900–750, respectively (Table 2). The ν_4_ (PO_4_^3−^) vibrational band shows two separate peaks at 604 and 565 cm^−1^ related to the atomic disorder and/or the crystalline size of bioapatite^68^. Variations in these properties among samples were monitored through the FW85% parameter. The FW85% parameter was calculated as the the width of the phosphate peak at 604 cm^−1^ measured at the 85% of the height with respect to the baseline, that is defined by two points calculated as the local minimum of the spectra in the ranges 850–620 and 510–470 cm^−1^^81^. FW85% is inversely related to the Splitting Factor (IRSF) that quantifies the extent of the splitting of the two peaks and calculated as the sum of the two peak intensities of the ν_4_ (PO_4_^3−^) mode divided by the intensity of the valley between them^62^. IRSF is widely used in the literature to monitor the degree of recrystallisation of the bioapatite: higher values of IRSF show a more ordered crystal structure with an increased crystal size,^109,110^. The mineral-to-matrix ratios were calculated by dividing the Amide I (1660 cm^−1^) intensity by the main phosphate peak intensity at 1035 cm^−1^ (ν_3_ (PO_4_^3−^)) (**AmI/PO**_**4**_^**3−**^). A more detailed description of the infrared parameters here calculated is reported in Scaggion et al.,^89^, and see Tables 2 and 3.

**Table 2:** Wavenumber and baselines adopted to calculate the peaks intensity and width of tooth and bone sample properties by FTIR spectroscopy.

Vibrational mode and functional group	Wavenumber (cm^−1^)	Baseline (cm^−1^)
AmideI ν (C=O) stretch	1660	2000–1800/1400–1200
ν_3_(PO_4_^3−^) antisymmetric stretching	1035	1400–1200/900–750
ν_4_(PO_4_^3−^) bend	604	850–620/510–470

**Table 3:** List of FTIR-based parameters and comparisons used.

Parameters	Wavenumber (cm^−1^)	Characterization
FW85%	Width at 85% of the height of the 604 cm^−1^ peak	Atomic order/disorder
AmI/PO_4_^3−^	1660 cm^−1^/1035 cm^−1^	Amount of amide on phosphate
1660:1690 cm^−1^ ratio	1660 cm^−1^/1690 cm^−1^	Collagen quality

### Lyophilized bone collagen spectra analyses

Collagen cross-links provide the fibrillar collagen matrices with properties such as tensile strength and viscoelasticity, as described in Paschalis et al.,^102^; these properties can be modified by diseases but also by interaction with the burial environment^73^. The ratio of peak areas of the mature non-reducible (interfibrillar) cross-links and immature reducible (intrafibrillar) cross-links of Amide I sub-underlying of ∼1660 and ∼1690 cm^−1^ (**1660:1690**) provides a semi-quantitative measure of the enzymatic cross-linking in the organic matrix. According to the literature^102^, the degree of cross-linking of collagen from immature to mature has been calculated from 1660:1690 cm^−1^ area ratio which informs on the collagen maturity. Most of the scientific community considers this a valid parameter^73,111–113^ that represents collagen maturity, although its validation is still controversial, especially with respect to the overlap of structural water absorption interference^85,114^ at ~ 1645 cm^−1^ (H–O–H bending). For our study, this parameter, which can be considered an indicator of the degree of collagen alteration^111^, was estimated by employing a combination of second-derivative spectra and peak finding to determine the underlying peaks of Amide I region at ~ 1660 and ~ 1690 cm^−1^^115–117^. The sub-underlying area ratio (1660:1690) was calculated for all collagen samples.

**Figure 5:**
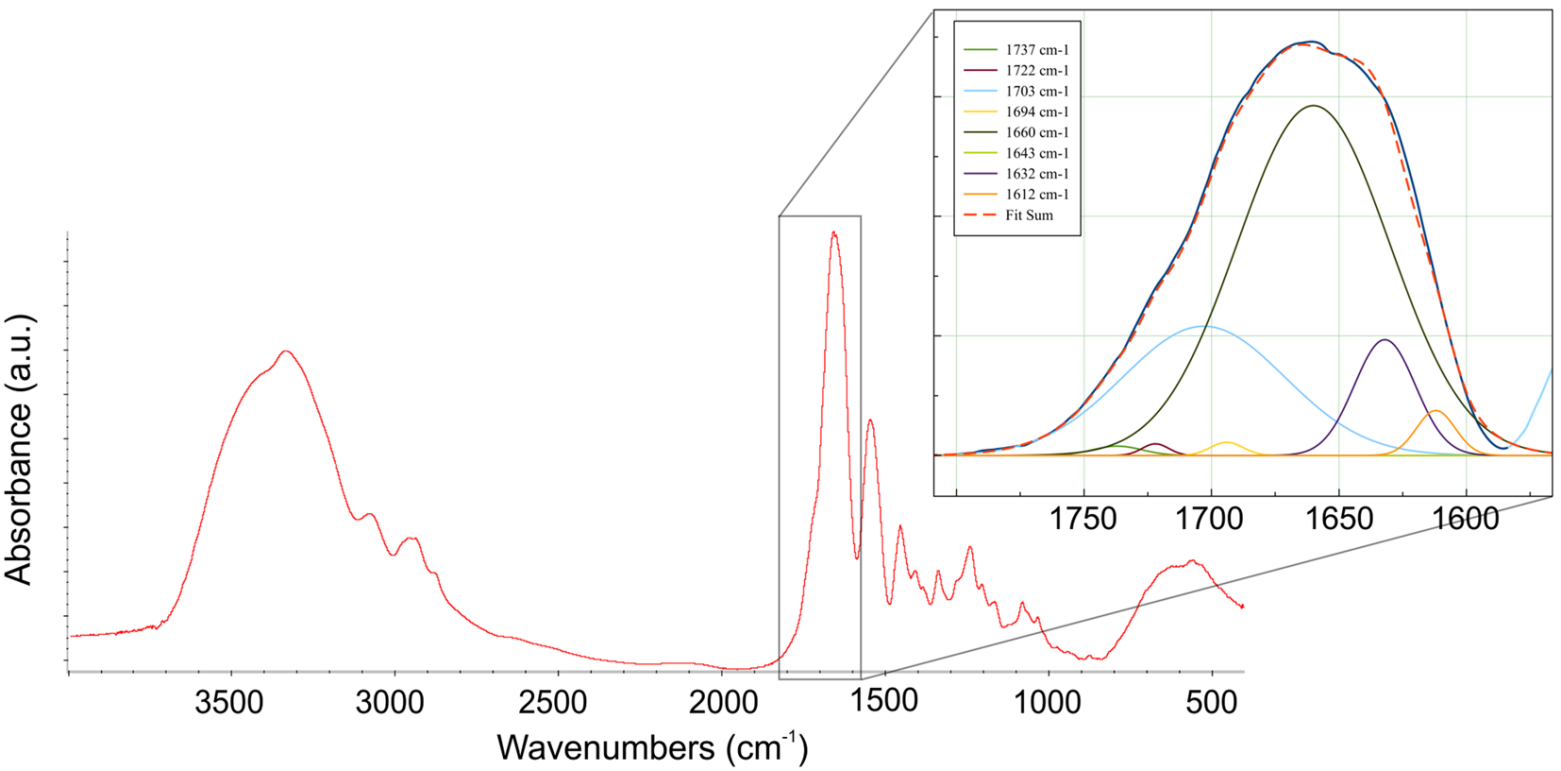
Example of spectral deconvolution of Amide I band calculated for each collagen spectra. Characteristic sub-bands are reported in figure.

The presence of sub-underlying signals of Amide I to calculate the denaturation state of collagen has been estimated by employing the second-derivative analysis (calculated using Savitsky-Golay smoothing technique with 9 smoothing points)^118^. OMNIC^Ⓡ^ software was used to convert the FTIR absorbance spectra into second derivatives to determine the number and position of components corresponding to the Amide I region for the next curve-fitting process of collagen spectra. For the average collagen spectrum, eight sub-components were identified at 1737–1722-1703–1694-1660–1643–1632–1612 cm^−1^. These sub-bands were chosen according to known peak position from literature^119–122^ (Fig. 5).

As for the curve fit, MagicPlot software was chosen for nonlinear fit, plotting and data analysis. For each collagen spectrum, a baseline was defined in the region between 1800–1300 cm^−1^ of the selected spectrum, and a Gaussian function was used for spectral deconvolution. The sub-components position was selected setting the peak width at 16 cm^−1^ (4 $\times$ the sensitivity of the tool) measuring the principal areas of ~ 1660 and ~ 1690 cm^−1^.

### Bone collagen extraction and isotopic analysis

500 mg of lyophilized bone collagen was extracted from 17 archaeological sub-samples and one fresh animal bone previously selected (Table S1). Sample preparation was carried out in the Mass Spectrometry lab of stable isotopes of the Department of Geosciences (University of Padova) using the standard laboratory protocol based on^123^ and^124^. After a meticulous cleaning, the bone fragments were demineralised in c. 10 ml 0.5 M aq. HCl and left in the fridge (4 °C) for several days. The samples were then gelatinised in pH 3 water solution at 75 °C in the oven for 48 h. Finally, the collagen was lyophilized before being weighed for isotopic analysis. Isotope analyses were performed using a Thermo Scientific Delta V Advantage Isotope Ratio Mass Spectrometer in continuous-flow mode coupled to a Flash 2000 Elemental Analyzer and a ConFlo IV interface. The isotopic composition of carbon (δ^13^C) and nitrogen (δ^15^N) was calibrated respectively on the VPDB and AIR scales, using international standards (CH-6 and CH-7 for carbon, N-1 and N-2 for nitrogen and both UREA). Isotope data were obtained for all collagen samples analysed by FTIR. Stable carbon (δ^13^C) and nitrogen (δ^15^N) isotopic analyses of collagen, preserved in subfossil and archaeological bone and tooth, are a powerful tool for the reconstruction of the environment and paleo-diet^125^ and there is a growing interest in protein available in hard tissue archives. The quality of measurements of carbon and nitrogen isotopes in ancient samples is confirmed by a robust protocol for the detection of contaminants and diagenesis^126^. The most widely used quality control (QC) criterion is the atomic ratio (C:N), for which ancient samples have an acceptable quality when ranging between 2.9 and 3.6^87^. According to Guiry and Szpak^127^, this QC method, also widely used on modern samples, is not directly transferable to analyses of fresh collagen, thus establishing a new range for mammals’ modern collagen between 3.00 and 3.28.

### Ancient DNA extraction

Minimally destructive sampling of ancient human remains was applied for 41 bone samples from Grottina Covoloni del Broion, Salapia, San Giovanni Rotondo and Ordona (Table S1) using protocols that will allow for different types of biomolecular or chemical analysis. The first layer of petrous bone was removed with a sterilised drill bite to avoid exogenous contamination and a core of 10 mm was extracted from the *cochlea*. The drill bits and core drill were sterilised in between samples with 6% (w/v) bleach followed by distilled water and then ethanol rinse, while the tooth roots were removed with a sterile drill wheel. The root and the petrous portions were soaked in 6% (w/v) bleach for 5 min. The samples were rinsed three times with 18.2 ddH_2_O and soaked in 70% (v/v) Ethanol for 2 min. Tubes were shaken during the procedure to dislodge particles. One the sample was weighed, 20 $\times$ ml of ETDA for sample mass and 0.5 $\times$ Proteinase K for sample mass were added into PCR-clean 5 ml or 15 ml conical tubes (Eppendorf) along with the samples inside the IIB hood and the tubes were incubated 72 h on a slow shaker at room temperature. The DNA extracts from bone samples were concentrated to 250 µl using the Vivaspin Turbo 15 (Sartorius) and purified in large volume columns (High Pure Viral Nucleic Acid Large Volume Kit, Roche) using 2.5 ml of PB buffer, 1 ml of PE buffer, and 100 µl of EB buffer (MinElute PCR Purification Kit, QIAGEN).

For the elution of the endogenous DNA, the silica columns were transferred to a collection tube to dry and followed in 1.5 ml DNA lo-bind tubes (Eppendorf) to elute. Samples were incubated with 100 µl EB buffer at 37 °C for 10 min and centrifuged at 13,000 rpm for 2 min. After centrifugation, the silica columns were removed, and the samples were stored at − 20 °C. Only one extraction per sample was performed for screening and 30 ml used for libraries. Laboratory extraction was performed in dedicated ancient DNA laboratories at the Estonian Biocentre, Institute of Genomics, University of Tartu, Tartu, Estonia. The library quantification and sequencing were performed at the Core Facility of the Institute of Genomics, Tartu, Estonia. Complete procedures and DNA analyses are detailed in Aneli et al.^30^ and Saupe et al.^31^.

### Statistical analysis

Linear regression analysis (lm), a fitting modelling technique that estimates the relationship between two or more variables^128,129^, was performed. The regression analysis was chosen because it allows one to highlight a relationship between one or more independent variables (IR parameters used) and the dependent one (%aDNA). The correlation coefficient of the multiple regression model (R-sq in Table 1) was used to evaluate the predictive potential of FTIR parameters for aDNA relative abundance. The model in R was created using aDNA values as the target variable and FW85% parameter as the predictive variable.

Linear regression (function *lm*) in R free statistical software (4.1.2 version, available at www.r-project.org) allows to model a continuous variable Y as a mathematical function of one or more X variables, so that it can be used the regression model with X known to predict the Y variable^130^.

$$\begin{gathered} {\text{Y}} = \beta_{{1}} + \beta_{{2}} {\text{X}} + \epsilon \hfill \\ \left( {\text{linear model formula}} \right) \hfill \\ \end{gathered}$$

β_1_ is the intercept and β_2_ is the slope and are called regression coefficients, ϵ is the error term.

The contribution of the FW85% parameter measured for each bone FTIR spectra was combined with endogenous aDNA yields to define a predictive model. Data were integrated with other 56 infrared measures reported in Scaggion et al.^89^ (Table S2) following the same procedures for sample preparation and analysis of the IR spectra of petrous bones and tooth roots. A set of femurs was also considered, once verified that different bone tissues show similar diagenetic alterations^89^, to enlarge the set of samples and to increase the statistics. An endogenous DNA yield of 50% and 100% was attributed to samples for which no genetic extraction was carried out, such as modern bones from the Tedeschi collection^131^ and fresh bones from animals of several taxa^81^. A null value was attributed to highly diagenised osteological samples from the archaeological site of Al-Khiday (Central Sudan)^132,133^ for which aDNA extraction was reported to have failed in previous attempts.

### Ethical statement

The human osteological remains, analysed for this manuscript, are preserved in the collections of the Museum of Nature and Humankind- Anthropology section, Padova University. Bone remains come from archaeological campaigns of necropolis and caves carried out during the nineteenth-twentieth century and from exhumations, cemetery ossuaries or are acquired by Hospitals or other charitable organizations, the latter mostly collected by Prof. Enrico Tedeschi (1860–1931). The Museum of Nature and Humankind- Anthropology section also houses an important ancient osteological collection of about 200 human skeletons coming from the archaeological site of Al-Khiday (Central Sudan) covering a wide chronological span (7000 BCE to the II c.CE). Other bone elements analyzed were provided by the Archaeological Superintendence of the Metropolitan Area of Venice and for the Provinces of Belluno, Padova and Treviso and by the Superintendence of Cultural Heritage of Trento.

### Supplementary Information

Supplementary Tables.

## Data Availability

The accession number for the DNA sequences reported in this paper is ENA:PRJEB37660 (https://www.ebi.ac.uk/ena/data/view/PRJEB37660) and ENA:PRJEB47831. Data are also available through the data depository of the EBC (http://evolbio.ut.ee) and of the EBC-IG (http://www.ebc.ee/free_data/AneliSaupe_2021/).
