# Supplementary material for: A fresh perspective on infrared spectroscopy as a prescreening method for molecular and stable isotopes analyses on ancient human bones
Source: Sci Rep. 2024 Jan 10;14:1028. doi: 10.1038/s41598-024-51518-5 (PMC10781948; doi:10.1038/s41598-024-51518-5)
Supplement: Supplementary file 1 — Supplementary Tables. [file 41598_2024_51518_MOESM1_ESM.pdf]

A fresh perspective on infrared spectroscopy as a prescreening method for molecular and stable isotopes analyses on ancient human bones

Cinzia Scaggion[1,2*], Maurizio Marinato[3], Gregorio Dal Sasso[4], Luca Nodari[5], Tina Saupe[6], Serena Aneli[7,8], Luca Pagani[6,7], Christiana L. Scheib[6], Manuel Rigo[1,4], Gilberto Artioli[1,2].

[1]Department of Geosciences, University of Padova, 35131, Padova, Italy.
[2]INSTM, National Interuniversity Consortium of Materials Science and Technology, 50121, Firenze, Italy.
[3]Department of Cultural Heritage: Archaeology and History of Art, Cinema and Music, University of Padova, 35139, Padova, Italy.
[4]Institute of Geosciences and Earth Resources, Italian National Research Council-CNR, 35131, Padova, Italy
[5]Institute of Condensed Matter Chemistry and Technologies for Energy, Italian National Research Council-CNR, 35127, Padova, Italy
[6]Estonian Biocentre, Institute of Genomics, University of Tartu, 51010, Tartu, Estonia
[7]Department of Biology, University of Padova, 35122, Padova, Italy
[8]Department of Public Health Sciences and Pediatrics, University of Torino, 10126, Torino, Italy

*corresponding author, e-mail address: cinzia.scaggion@phd.unipd.it (C. Scaggion)

Supplementary Information

Table of content

➢ Supplementary Table S1 p. 2
➢ Supplementary Table S2 p. 6

Supplementary Table S1

Sample	Chronology	Archaeological site	Skeletal element	C:N ratio	aDNA (%) endogenous	1660 cm^{-1}	16690 cm^{-1}	1660:1690 cm^{-1}	AmI/PO_4^{3-}	FW85%	IRSF
BR-03_ PbGCB O-Ce	Bronze Age	Grottina Covoloni Broion, Italy	petrous bone		5.44%				0.12	9.54	3.68
BR-04_PbGCB O-Ce	Bronze Age	Grottina Covoloni Broion, Italy	petrous bone		4.90%				0.14	10.36	3.41
BR-01_Pb GCB O:38	Bronze Age	Grottina Covoloni Broion, Italy	petrous bone		7.81%				0.11	9.57	3.66
BR-05_ PbGCB	Bronze Age	Grottina Covoloni Broion, Italy	petrous bone		7.14%				0.16	10.04	3.41
BR-25_GCB	Bronze Age	Grottina Covoloni Broion, Italy	tooth		0.09%				0.25	11.2	3.18
BR-06_GCB	Bronze Age	Grottina Covoloni Broion, Italy	tooth		0.88%				0.31	9.67	3.56
BR-07_GCB	Bronze Age	Grottina Covoloni Broion, Italy	tooth		0.13%				0.35	12.02	3.00
BR-09_GCB	Bronze Age	Grottina Covoloni Broion, Italy	tooth		0.13%				0.13	11.54	3.53
BR-16_ GCB 4B+4S 5S	Bronze Age	Grottina Covoloni Broion, Italy	tooth		0.42%				0.31	9.65	3.10
BR-22_ GCB 7-353	Bronze Age	Grottina Covoloni Broion, Italy	tooth	3.4	0.07%	15.8	10.0	1.57	0.11	9.39	3.64
BR-24_ GCB 53	Bronze Age	Grottina Covoloni Broion, Italy	tooth		3.86%				0.40	11.97	3.09
BR-10_GCB 223	Bronze Age	Grottina Covoloni	tooth		0.30%				0.32	9.41	3.70

		Broion, Italy									
BR-27_GCB 275	Bronze Age	Grottina Covoloni Broion, Italy	tooth		0.22%				0.38	12.24	2.99
BR-13_ GCB274	Bronze Age	Grottina Covoloni Broion, Italy	tooth		0.25%				0.37	12.25	2.99
BR-17_GCB 629	Bronze Age	Grottina Covoloni Broion, Italy	tooth		0.35%				0.17	9.98	3.29
BR-21_GCB 807	Bronze Age	Grottina Covoloni Broion, Italy	tooth		1.52%				0.24	9.4	3.76
BR-19_ GCB 909	Bronze Age	Grottina Covoloni Broion, Italy	tooth	3.3	17.32%	15.8	10.0	1.57	0.17	10.12	3.37
BR-12_ GCB O:156	Bronze Age	Grottina Covoloni Broion, Italy	tooth		0.70%				0.38	11.8	3.11
BR-14_ GCB SeH6 Str248	Bronze Age	Grottina Covoloni Broion, Italy	tooth	3.2	9.40%	15.8	10.4	1.51	0.36	11.74	3.05
BR-18_GCB 221	Bronze Age	Grottina Covoloni Broion, Italy	tooth		0.85%				0.35	11.28	3.13
ORD001_PB	Iron Age	Ordona. Italy	petrous bone	3.3	2.14%	12.0	7.6	1.58	0.08	9.09	4.07
ORD004_PB	Iron Age	Ordona. Italy	petrous bone	3.1	5.50%	14.4	9.3	1.54	0.10	10.39	3.87
ORD006_PB	Iron Age	Ordona. Italy	petrous bone	3.3	31.42%	9.2	6.2	1.48	0.05	9.43	4.39
ORD009_PB	Iron Age	Ordona. Italy	petrous bone	3.1	42.27%	11.2	7.5	1.49	0.05	9.75	4.61
ORD010_PB	11^{th}c.AD	Ordona. Italy	petrous bone	3.4	43.75%	7.1	5.2	1.36	0.14	9.59	4.16

ORD011_PB	Iron Age	Ordona. Italy	petrous bone	3.3	14.73%	8.2	5.5	1.51	0.19	10.78	3.51
ORD012_PB	Iron Age	Ordona. Italy	petrous bone		0.09%				0.05	9.16	4.36
ORD014_PB	Iron Age	Ordona. Italy	petrous bone	3.2	15.55%	11.7	7.8	1.50	0.11	9.04	4.37
ORD018_PB	Iron Age	Ordona. Italy	petrous bone		0.69%				0.06	9.23	4.13
ORD019_PB	Iron Age	Ordona. Italy	petrous bone		3.69%				0.06	9.7	4.29
SAL001_T	Iron Age	Salapia. Italy	tooth		4.71%				0.18	9.58	3.73
SAL003_PB	Iron Age	Salapia. Italy	petrous bone	3.3	28.16%	9.8	6.6	1.49	0.12	9.94	4.11
SAL005_PB	Iron Age	Salapia. Italy	petrous bone		0.99%				0.11	9.59	4.45
SAL007_PB	Iron Age	Salapia. Italy	petrous bone		3.39%				0.11	8.67	5.13
SAL008_T	Iron Age	Salapia. Italy	tooth		1.31%				0.23	9.99	3.67
SAL010_PB	Iron Age	Salapia. Italy	petrous bone		2.74%				0.04	8.96	5.12
SAL011_T	Iron Age	Salapia. Italy	tooth	3.1	3.85%	10.0	6.1	1.65	0.40	11.44	3.22
SAL012_T	Iron Age	Salapia. Italy	tooth		0.15%				0.07	9.12	4.58
SGR001_T	8thc.AD	San Giovanni Rotondo. Italy	tooth	3.1	3.72%	13.5	8.4	1.61	0.18	10.41	3.47
SGR002_T	Iron Age	San Giovanni Rotondo. Italy	tooth		17.79%				0.10	9.28	3.81
SGR003_T	Iron Age	San Giovanni	tooth		6.39%				0.27	11.44	3.27

		Rotondo. Italy									
oxbone.SPA	21st c.AD	Fresh bone	femur	3		5.6	2.8	1.99	0.39	12.51	3.01
DSL17D	Middle Age (VII-IX c.AD)	Desenzano del Garda. Italy	petrous bone	2		3.0	2.6	1.16	0.08	9.04	4.29
DSL17B	Middle Age (VII-IX c.AD)	Desenzano del Garda. Italy	tooth	5.6		7.9	6.6	1.19	0.22	9.36	3.64
DSL25	Middle Age (VII-IX c.AD)	Desenzano del Garda. Italy	tooth	4		7.0	5.8	1.20	0.33	11.21	3.14

Table S1. *Information on the samples used for the statistical model and spectroscopic, C:N ratio and genetic results*

Supplementary Table S2

Sample	Chronology	Archaeological site	Skeletal element	aDNA (%) endogenous	AmI/PO_4^{3-}	FW85%	IRSF
0S1242	19th c.AD	Tedeschi collection	petrous bone	50%	0.44	13.25	2.87
0S419	19th c.AD	Tedeschi collection	petrous bone	50%	0.50	12.09	3.03
0S426	19th c.AD	Tedeschi collection	petrous bone	50%	0.39	13.45	3.11
0S428	19th c.AD	Tedeschi collection	petrous bone	50%	0.31	11.50	3.21
0S431	19th c.AD	Tedeschi collection	petrous bone	50%	0.35	13.22	3.06
0S419	19th c.AD	Tedeschi collection	tooth	50%	0.40	11.57	3.24
0S426	19th c.AD	Tedeschi collection	tooth	50%	0.39	11.97	3.17
0S428	19th c.AD	Tedeschi collection	tooth	50%	0.37	12.35	3.09
0S431	19th c.AD	Tedeschi collection	tooth	50%	0.38	11.76	3.23
0S1453	19th c.AD	Tedeschi collection	tooth	50%	0.35	13.67	3.05
3F-gr2	Neolithic	Al-Khiday	femur	0%	0.12	8.45	4.71
31F-gr3	Mesolithic (?)	Al-Khiday	femur	0%	0.05	7.64	5.64
50F-gr5	Meroitic	Al-Khiday	femur	0%	0.04	7.12	6.17
55-gr2	pre-Mesolitic	Al-Khiday	femur	0%	0.08	8.59	3.74
103-gr3	Neolithic	Al-Khiday	femur	0%	0.06	7.74	4.78
115F-gr4	Meroitic	Al-Khiday	femur	0%	0.04	6.72	6.24
128-gr5	Meroitic	Al-Khiday	femur	0%	0.06	8.61	4.60
136-gr5	Meroitic	Al-Khiday	femur	0%	0.04	6.77	6.33
156-gr5	Meroitic	Al-Khiday	femur	0%	0.06	8.55	4.00
158-gr4	Meroitic	Al-Khiday	femur	0%	0.06	8.90	3.90

159F-gr3	pre-Mesolitic	Al-Khiday	femur	0%	0.11	8.27	4.65
163-gr5	pre-Mesolitic	Al-Khiday	femur	0%	0.03	6.82	6.07
170-gr5	pre-Mesolitic	Al-Khiday	femur	0%	0.03	7.91	4.68
177-gr4	pre-Mesolitic	Al-Khiday	femur	0%	0.06	8.48	3.35
Al-K_12T	pre-Mesolitic	Al-Khiday	tooth root	0%	0.05	8.91	3.83
Al-K_34T	Neolithic	Al-Khiday	tooth root	0%	0.02	7.53	5.65
Al-K_35T	pre-Mesolitic	Al-Khiday	tooth root	0%	0.05	8.65	3.85
Al-K_58T	Meroitic	Al-Khiday	tooth root	0%	0.03	8.21	5.46
Al-K_95T	Neolithic	Al-Khiday	tooth root	0%	0.02	7.53	5.76
Al-K_104T	Neolithic	Al-Khiday	tooth root	0%	0.02	7.93	4.90
Al-K_163T	Mesolithic (?)	Al-Khiday	tooth root	0%	0.03	7.66	5.47
Al-K_164T	Mesolithic (?)	Al-Khiday	tooth root	0%	0.03	7.47	5.35
Al-K185T	pre-Mesolitic	Al-Khiday	tooth root	0%	0.05	9.13	3.47
Al-K_186T	Meroitic	Al-Khiday	tooth root	0%	0.04	8.36	5.28
Al-K_187T	Meroitic	Al-Khiday	tooth root	0%	0.03	7.46	5.77
Al-K_12PB	pre-Mesolitic	Al-Khiday	petrous bone	0%	0.04	9.34	3.89
Al-K_34PB	Neolithic	Al-Khiday	petrous bone	0%	0.06	8.11	5.53
Al-K_35PB	pre-Mesolitic	Al-Khiday	petrous bone	0%	0.06	9.27	3.62
Al-K_58PB	Meroitic	Al-Khiday	petrous bone	0%	0.02	8.45	5.75
Al-K_95PB	Neolithic	Al-Khiday	petrous bone	0%	0.02	7.62	5.79
Al-K104PB	Neolithic	Al-Khiday	petrous bone	0%	0.07	8.08	4.33
Al-K163PB	Mesolithic (?)	Al-Khiday	petrous bone	0%	0.03	8.03	5.28

Al-K164PB	Mesolithic (?)	Al-Khiday	petrous bone	0%	0.02	7.53	5.87
Al-K185PB	pre-Mesolitic	Al-Khiday	petrous bone	0%	0.04	9.27	3.57
Al-K186PB	Meroitic	Al-Khiday	petrous bone	0%	0.03	8.44	5.79
Al-K187PB	Meroitic	Al-Khiday	petrous bone	0%	0.03	8.19	5.12
Dog08	21st c.AD	Fresh bone	femur	100%	0.44	12.93	3.04
Horse10	21st c.AD	Fresh bone	femur	100%	0.62	16.09	2.82
O.sheep11	21st c.AD	Fresh bone	femur	100%	0.39	13.13	2.99
Pig10	21st c.AD	Fresh bone	femur	100%	0.64	17.60	2.85
Y.sheep06	21st c.AD	Fresh bone	tooth root	100%	0.39	14.34	2.85
O.sheep05	21st c.AD	Fresh bone	tooth root	100%	0.32	12.23	3.05
O.sheep06	21st c.AD	Fresh bone	tooth root	100%	0.32	12.40	3.04
Horse07	21st c.AD	Fresh bone	tooth root	100%	0.36	14.33	2.77
Human25	21st c.AD	Fresh bone	tooth root	100%	0.37	13.34	2.99

Table S2. *A set of samples including femurs come from Al-Khiday, modern and fresh bones used for the statistical model are also reported in Scaggion et al.2024* [91].
